# Coastal erosion as a source of mercury into the marine environment along the Polish Baltic shore

**DOI:** 10.1007/s11356-016-6753-7

**Published:** 2016-05-10

**Authors:** Magdalena Bełdowska, Agnieszka Jędruch, Leszek Łęczyński, Dominika Saniewska, Urszula Kwasigroch

**Affiliations:** Institute of Oceanography, University of Gdańsk, Piłsudskiego 46, 81-378 Gdynia, Poland

**Keywords:** Airborne LiDAR, Coastal abrasion, Cliff, Climate change, Extreme phenomena, Mercury

## Abstract

The climate changes in recent years in the southern Baltic have been resulting in an increased frequency of natural extreme phenomena (i.e. storms, floods) and intensification of abrasion processes, which leads to introduction of large amounts of sedimentary deposits into the marine environment. The aim of this study was to determine the mercury load introduced to the Baltic Sea with deposits crumbling off the cliffs—parts of the coast that are the most exposed to abrasion. The studies were carried out close to five cliffs located on the Polish coast in the years 2011–2014. The results show that coastal erosion could be an important Hg source into the marine environment. This process is the third most important route, after riverine and precipitation input, by which Hg may enter the Gulf of Gdańsk. In the Hg budget in the gulf, the load caused by erosion (14.3 kg a^−1^) accounted for 80 % of the wet deposition and was 50 % higher than the amount of mercury introduced with dry deposition. Although the Hg concentration in the cliff deposits was similar to the natural background, due to their large mass, this problem could be significant. In addition, the preliminary studies on the impact of coastal erosion on the Hg level in the marine ecosystem have shown that this process may be one of the Hg sources into the trophic chain.

**Electronic supplementary material:**

The online version of this article (doi:10.1007/s11356-016-6753-7) contains supplementary material, which is available to authorized users.

## Introduction

Mercury (Hg) is one of the most dangerous global pollutants. The adverse impact of Hg on the environment is related to its strong chemical and biological activity, as a result of which it is easily absorbed by organisms and spreads in the environment very rapidly. The Hg compounds become accumulated in tissues and can undergo biomagnification in organisms on higher trophic levels, reaching concentrations many times higher than in the environment itself (Förstner and Wittman 1981). Even small amounts of Hg in the system may lead to the disruption of biochemical processes, and it is also known to interfere with enzymatic and hormonal reactions, as well as protein and lipid biosynthesis (Carocci et al. 2014; Rice et al. 2014). Hg is a neurotoxin that leads to impairments in the human nervous system—autism and learning difficulties in children. In adults, it is associated with the Alzheimer’s and Parkinson’s diseases, as well as depression. Hg penetrates easily through the uterus barrier, resulting in miscarriages and embryo impairments. Owing to the fact that the main route of Hg introduction into the human body is the consumption of fish and seafood, investigating and determining the sources of this compound in the marine ecosystem are of great importance (Bose-O’Reilly et al. 2010).

Hg is introduced to the Baltic Sea mainly through rivers transporting pollutants from their respective drainage areas, but also through atmospheric deposition (Bełdowska et al. 2014b). An important role in the Hg balance in a water basin is also played by the remobilisation of the metal accumulated in bottom sediments (Bełdowski and Pempkowiak 2007; Bełdowski et al. 2009). Additional loads of Hg are introduced into the Baltic Sea with water flowing in from the North Sea and with the submarine groundwater discharge or as a result of sinking debris or ships (Wrembel 1993; Szefer 2002; Szymczycha et al. 2013). Given that Hg has a highly adverse effect on the environment, in recent years, a number of activities have been undertaken in order to reduce its emission into the basin (i.e. reduction of Hg supply and demand for its use in industrial processes, control of international trade of Hg, environmentally safe waste management or remediation of Hg-contaminated sites) (HELCOM 2010). The problem of Hg pollution has been included in many international conventions and agreements such as the UNEP Global Mercury Partnership (GMP), the HELCOM Baltic Sea Action Plan (BSAP), as well as the European Union directives, i.e. Integrated Pollution Prevention and Control (IPPC) or Water Framework Directive (WFD). Since restrictions were implemented in the 1990s, the load of Hg entering the Baltic Sea has decreased by 44 % and, of the total amount, it is currently estimated that about 70 % of Hg reaching the sea comes from anthropogenic sources (Bartnicki et al. 2012; HELCOM 2010). However, in addition to direct human activity, advancing climate changes also have an important influence on the Hg load introduced to the basin, as well as on its mobility and transformations in the marine environment (Bełdowska 2015; Bełdowska et al. 2016). A warm winter undoubtedly contributes to a significant reduction in Hg dry deposition fluxes which are associated with reduction of coal combustion in the heating season—the main source of Hg to the atmosphere (Bełdowska et al. 2014b; Bełdowska 2015). On the other hand, the extreme phenomena such as floods also contribute to the greater Hg remobilisation per annum, generally leaching historical land-based deposits (Saniewska et al. 2014b). Such processes are of major significance to marine organisms inhabiting the coastal zone and estuaries, as they cause toxic substances to be introduced into the sea within a relatively short time. Frequent storms and the rising level of the sea play a decisive role in determining the shaping tendency of the coastline. As they become more intense, there is also an increase in the processes of coastal erosion (i.e. cliff abrasion) or processes such as land sliding, fall-off and wash-off (Cieśliński and Chromiak 2010; Jania and Zwoliński 2011). It is estimated that more than half of the Polish Baltic coast undergoes intense erosion processes, and the average retreat rate of the Polish coastline (1 m a^−1^) is now twice as high as in the years 1960–1980 (Dubrawski and Zawadzka-Kahlau 2006). The fine deposit material that is formed in this way is then transported by waves to regions with lower environmental dynamics, such as the deeper areas of the basin (Basiński 1993).

Dynamic and evolution of the southern Baltic coastline have been the subject of numerous studies for many years (i.e. Rotnicki et al. 1995; Subotowicz 1982, 1995; Zawadzka-Kahlau 1999; Mojski 2000). However, there are no studies on the effect of coastal erosion on the load of pollutants introduced to the marine environment in this way. Recognizing this problem is particularly important—nowadays, a lot of attention is given to reducing Hg emission into the marine environment; on the other hand, intensification of coastal erosion may lead to increase of the Hg load. The aim of the present studies, conducted within the framework of the National Science Center project No. UMO 2011/01/B/ST10/07697, was to determine the influence of cliff abrasion on the load of Hg introduced to the Polish part of the Baltic Sea.

## Materials and methods

### Study area

**Fig. 1:**
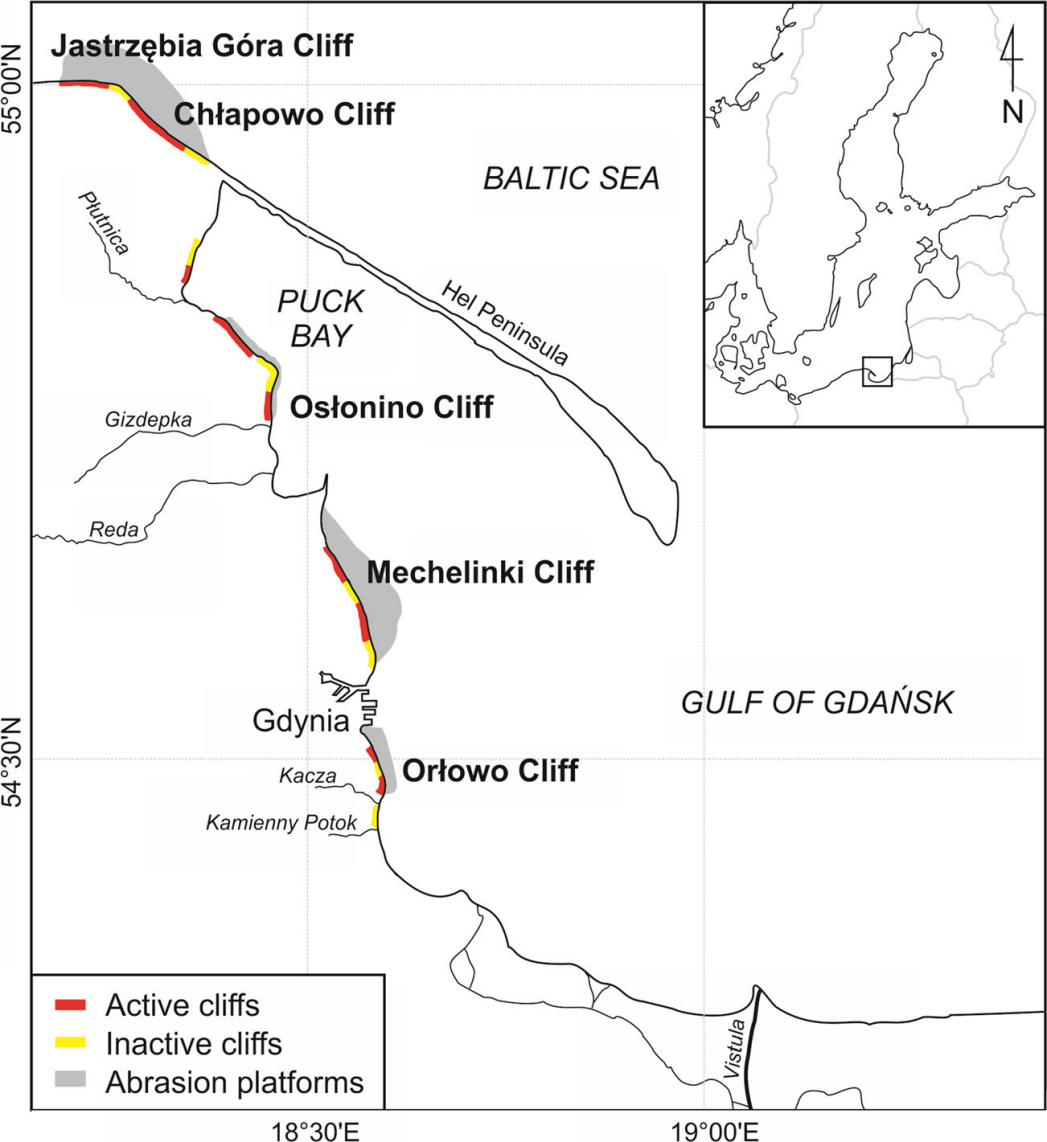
Study area including geomorphology of the southern Baltic coast (based on Subotowicz 1980; modified by the authors)

Studies were carried out on five cliff sections along the Polish coast (Fig. 1, Table 1). Three of these (the cliffs at Orłowo, Mechelinki and Osłonino) are located in the western part of the Gulf of Gdańsk, while the remaining two (the cliffs at Chłapowo and Jastrzębia Góra) are situated on the open part of the southern Baltic.

**Table 1:** Characteristic of the selected cliffs located in the Polish shore (based on Dubrawski and Zawadzka-Kahlau 2006) and the annual loads of deposits and Hg_TOT_ introduced into southern Baltic as a result of coastal erosion in years 2011–2014

Cliff	Localization at the Polish coastline (km–km)	Length (km)	Height (m)	Deposits load (t a^−1^)	Hg_TOT_ load (kg a^−1^)
Orłowo	81.30–81.95	0.65	15–44	9647	0.08
Mechelinki	95.65–95.90	0.25	25–30	20 076	0.18
Osłonino	107.35–107.75	0.40	15	8575	0.08
Chłapowo	126.75–130.70	3.95	30–50	218,254	1.92
Jastrzębia Góra	131.70–134.50	2.80	30–35	106,119	0.93

The eroded sections at present cover about 147 km of the Polish coast, which amounts to 30 % of its length (Uścinowicz et al. 2004). They are dispersed along the majority of the coast, stretching along the coastline in sections reaching up to 10 km in length. The cliffs emerged in a coast made up of moraine elevations, which was eroded by the sea in the period of the Littorina Transgression (Subotowicz 1982; Tomczak 1992). The cliff coastline of the southern Baltic is comprised mainly of Pleistocene glacial tills (boulder clays) and fluvioglacial sands. Formations older than quaternary, such as loams or carbonate inserts, are less common. Occasionally, newer formations, which are composed of, for example, peats, gyttjas or eolic sands, can be found in the cliff structure (Subotowicz 1982; Uścinowicz et al. 2004). The Orłowo cliff is composed of two levels of till from the glacistadial of the main glaciation period in central Poland, while in the northern part of the cliff, there are Miocene sand-loam formations. Landslides sometimes occur there, as well as crumbling and fall-offs (Subotowicz 1982). The cliffs at Mechelinki and the Osłonino are mainly composed of sand-clay formations of glacial and fluvioglacial origin (Subotowicz 1971). The Hel Peninsula largely protects them from the effect of waves, and their activity is therefore much lower. The Chłapowo cliff is made of tills and fluvioglacial sand-gravel series, with formations of a tertiary base underneath, and is subject to developing slide and fall-off processes. The cliff in Jastrzębia Góra is made up of formations from the last glaciation period: layers of loam shales, separated by sand and gravels, whereas the slides are predominant morphodynamic process (Zawadzka-Kahlau 1994) (Fig. 1, Table 1).

### Sample collection

Samples of deposits, soils and plant material within the colluvium of the studied cliffs were collected manually in the years 2011–2014. In the Orłowo and Osłonino cliff areas, the studies were carried out in the years 2011–2013, during which time samples were collected 26 and 20 times, respectively. In the case of the Mechelinki, Chłapowo and Jastrzębia Góra cliffs, the samples were collected four times in the years 2013–2014. Each time, samples from seven to ten different places were collected within the area of each cliff. The collected deposits were transferred to polyethylene zip-lock bags and kept at −20 °C until analysis. Prior to total mercury (Hg_TOT_) analysis, the collected material was lyophilised then homogenised in a porcelain mortar. Additionally, a sub-sample of main type of deposits (boulder clay and sand) was collected in order to perform an analysis of its physical properties—loss of ignition and granulometric composition.

Additionally, throughout 2012, monthly sea water samples were collected at the bases of the Orłowo and Osłonino cliffs, with a further of five samples being taken just after crumbling (in December 2011, January 2012, March 2012, October 2012 and November 2012). In 2012, samples of water from rivers flowing into the Gulf of Gdańsk were also collected in order to analyse the concentration of Hg_TOT_. Samples of river waters were taken from river cross-section close to mouth, in which the impact of the sea was not observed: Vistula (12.0 km from the mouth), the Reda (2.6 km from the mouth), the Kacza River (0.1 km from the mouth) and the Gizdepka River (0.6 km from the mouth) (Fig. 1). Precipitation samples were also collected in 2012 using an automatic collector of wet deposition situated at the station in Gdynia (Fig. 1). The collection and storage methods of river waters and precipitation samples were described in an earlier study by Bełdowska et al. (2014b).

### Laboratory analysis

Hg_TOT_ concentration in the solid samples (deposits, soils and plants) was assayed in triplicates using atomic absorption spectrometry (AAS) on AMA 254 (Altec Ltd., Czech Republic). The quality control of the method involved an analysis of the certified reference material in three replications (GBW 07314—offshore sediment). The method was characterised by a high rate of recovery (98 %), and relative standard deviation did not exceed 5 %. Limit of detection (LOD) was 0.01 ng g^−1^. The analyses of Hg_TOT_ concentrations in liquid were carried out in the Department of Marine Chemistry and Biochemistry at the Institute of Oceanology, Polish Academy of Sciences (Sopot, Poland). Water samples for Hg analysis were oxidised by the addition of BrCl and pre-reduced with hydroxylamine hydrochloride solution 1 h prior to the analysis by cold vapour atomic fluorescence spectrometry (CVAFS) on TEKRAN 2600 (Canada), according to US EPA method 1631 (US EPA 2002). Quality control procedures for water samples included the use of blanks and deionized water spiked with mercury nitrate within the range of 0.5–25 ng L^−1^ and produced adequate precision (1 % RSD) and recovery (98–99 %). Quality control procedures involved an analysis of the certified reference material in three replications (BCR 579—coastal sea water) and revealed that the measurement uncertainty was below 5 %, while the LOD was as low as 0.05 ng L^−1^.

The estimation of the organic matter content in deposits and soils was determined using the loss on ignition method (LOI) at 550 °C (Santisteban et al. 2004), accepted as optimal for Baltic Sea sediments (Ciborowski 2010). Granulometric analysis were performed in order to determine the granulation of the sedimentary deposits, using the sieve method, which separated the particular sediment fractions through mechanical sifting on normalised sieves with net sizes of 2, 1, 0.5, 0.25, 0.125 and 0.063 mm (Myślińska 1992).

### Determination of Hg load

#### Coastal erosion

The load of sedimentary material introduced into the marine environment as a result of the coastal erosion was calculated by analysis of the temporal changes in the active layer of deposits occurring on the surface of the slopes of the Orłowo, Mechelinki, Osłonino, Chłapowo and Jastrzębia Góra cliffs in years 2009–2013. The basic material was the result of an airborne laser scanning (ALS) of the selected section of the coastline (Table 1), and the data of ALS were obtained courtesy of the Maritime Office (Gdynia, Poland). In recent years, the materials derived from the airborne laser scanning, i.e. light detection and ranging (LiDAR) which are among the most accurate data, gained importance. The results of numerous studies conducted around the world has shown that airborne LiDAR data are the key element of the monitoring of the shore development (i.e. Brock and Purkis 2009; Young and Ashford 2006; Kaczmarek 2015). Importantly, this method can be applied to the investigation of the shoreline recession of the soft-sediment as well as the rocky coast environments (Crapoulet et al. 2012; Earlie et al. 2015; Obu et al. 2016).

The LiDAR surveys were carried out by the APEX engineering company and were obtained for each site for 2009 and 2013, providing ca. 4-year period within which to analyse any change. In order to evaluate temporal changes along the cliffs, digital elevation models (DEMs) derived from the LiDAR surveys in raster format were used (point density: 7 points per m^2^). The DEMs have a lateral resolution of ±0.20 m and vertical resolution of ±0.15 m.

All surveys were preceded by an appropriate geometric calibration basing on reference terrain points, where the vertical and lateral resolutions were higher than 5 cm. Each result was also referred to the current sea state. Points positions were projected in two coordinate systems, both referring to the WGS 84 ellipsoid: UTM (33 and 34 N) and PUWG 1992. Collected data were interpolated afterwards and results were converted to create contour maps of selected cliff coasts. Area of interpolation was assigned respectively to reference publications considering the range and occurrence of certain cliff sections (Table 1, Online Resource 1). The first step of interpolation was to create regular grid with a true resolution of 3 m × 3 m applying the *SURFER 12* programme and its “kriging” method feature for this purpose. The next step was to mask area of interests with the “grid + blank” method. The same process was repeated for datasets collected in different time periods to create topography maps for each sampling. Afterwards, estimated cliff slope surfaces were compared by subtracting one elevation model from another to establish sedimentary material removal patterns (Online Resource 2) (Earlie et al. 2013; Earlie et al. 2015).

Calculating the difference between these two values enabled the determination of annual changes in the volume of active layers of colluvial sediments of the cliffs as a result of coastal erosion processes (net volumetric erosion) (Young and Ashford 2006). The mass of crumbling deposits was calculated with constant grain density of 2.65 g cm^−3^—a typical value for clays, sandy clays and clay sands, according to Polish Standards PN-88/B-04481 and PN-81/B-03020 (Myślińska 1992).

#### Riverine and atmospheric deposition input

The Hg load introduced to the southern Baltic Sea via rivers and precipitation was calculated in accordance with the methods described in earlier studies by Bełdowska et al. (2014b) and Saniewska et al. (2014a). Hg concentration in river water at cross sections close to the river mouth was used to calculate annual load of Hg transported into the sea, assuming linear variability in time periods between measurements (Niemirycz 2011):

$$ Lr={\displaystyle \sum_{i=1}^n{C}_i{Q}_i} $$

where *L*_*r*_—annual load (kg a^−1^); *n*—number of measurements; *C*_*i*_—discrete concentration of constituent in the *i*th measurement (μg m^−3^); *Q*_*i*_—discrete (daily) flow corresponding to the concentration *C*_*i*_ (m^3^ s^−1^).

The average daily flows of the Vistula and the Reda rivers were calculated according to National Environmental Monitoring standards by ADCP (WIOŚ 2011), while the average flows of the other studied rivers (Kacza and Gizdepka) were taken from the literature (Krajewska and Bogdanowicz 2009). Hg concentration in precipitation was used to calculate wet deposition fluxes:

$$ {F}_{wet}=CR $$

where *F*_*wet*_—wet deposition flux (ng m^−2^); *C*—Hg concentration in precipitation (ng L^−1^); *R*—amount of precipitation (mm = L m^−2^).

The precipitation amount during the experiments was measured by Huger Weather Station situated on the roof of the Institute of Oceanography, University of Gdańsk (Gdynia, Poland).

### Processing results

Statistical analysis and graphic representation of the obtained results were carried out using the *STATISTICA 10* programme by StatSoft. Owing to the fact that the number of samples was limited, the analysed data were not characterised by normal distribution (Shapiro–Wilk test *p* < 0.05). In order to determine the significance of differences, the non-parametric Kruskal–Wallis test was used. The dependencies between the analysed variables were determined on the basis of the Spearman’s coefficient, with a confidence interval of 95 %. The results of the granulometric examination of the deposits from the cliffs were processed using the *GRADISTAT 5.11* programme (Blott and Pye 2001). The minimum and maximum sizes of diameters within each sediment fraction were determined on the basis of Udden’s classification (Udden 1914), modified by Wentworth (1922). The map of the study area with the distribution of sampling stations was created using the *ArcGIS 10.1* programme by ESRI in the WGS 84 coordinate system.

## Results and discussion

The study material collected between 2011 and 2014 near the colluvium of the Orłowo, Mechelinki, Osłonino, Chłapowo and Jastrzębia Góra cliffs consisted of 542 samples of clastic material and 81 samples of plant material. The research material consisted of samples that varied lithologically—the following deposits types were identified: post-glacial boulder clay and fluvioglacial sands, as well as organogenic sediments—peat and soil. These types of deposits were typical for the cliffs of the Polish coast (Subotowicz 1980). The biological material consisted of various terrestrial plant remains: roots, leaves and grass.

### Characteristic of the sediments

Collected sediment samples were characterized by different granulometric composition and organic matter content. Samples of boulder clays (*n* = 304) were characterized by moderate sorting. Sediment grain size composition was dominated by the very fine sand (particle diameter 0.063–0.125 mm), whose percentage ranged from 8.3 to 44.2 % (mean 22.0 %, median 20.7 %), as well as the fine sand fraction (particle size 0.125–0.25 mm), the content of which accounted from 10.5 to 27.6 % (mean 20.3 %, median 18.9 %). A significant part in the composition of the particle size fraction was finely dispersed muddy sediments (particle diameter <0.063 mm), whose percentage ranged from 10.4 to 61.7 % (mean 19.0 %, median 17.2 %). The LOI values in boulder clays ranged from 1.45 to 11.09 % (mean 3.06 %; median 2.83 %). Fluvioglacial sand samples (*n* = 145) had poor or moderate sorting. The granulometric composition of these sediments accounted for the largest content of medium sand fraction (particle diameter 0.25–0.5 mm), whose percentage ranged from 4.8 to 69.3 % (mean 30.1 %, median 26.4 %), as well as the coarse sand fraction (particle diameter 0.5– 1 mm), whose content ranged from 14.0 to 47.5 % (mean 24.2 %, median 20.6 %). The percentage of finely dispersed sediments (particle diameter <0.063 mm) in the fluvioglacial sands does not exceed 10 % (mean 5.3 %, median 6.9 %). Loss of ignition in sands ranged from 0.12 to 1.17 % (mean 0.36 %, median 0.25 %).

### Hg concentration in the slide material of the cliffs

In all the analysed samples of deposit material, the concentrations of Hg_TOT_ occurred above the limit of detection (LOD). The data were not normally distributed (Shapiro–Wilk test, *p* = 0.00), while the obtained values ranged from 0.1 to 67.9 ng g^−1^ dw (*n* = 542, mean 9.0 ng g^−1^ dw, median 8.4 ng g^−1^ dw). The Hg_TOT_ concentrations were relatively low—similar to the natural background concentrations of mercury in the deposits occurring in the Baltic Sea region (Korhonen et al. 2001; Liepe et al. 2013) and several times lower than the Hg_TOT_ level in the surface bottom sediments of the southern Baltic Sea (Jędruch et al. 2015).

**Fig. 2:**
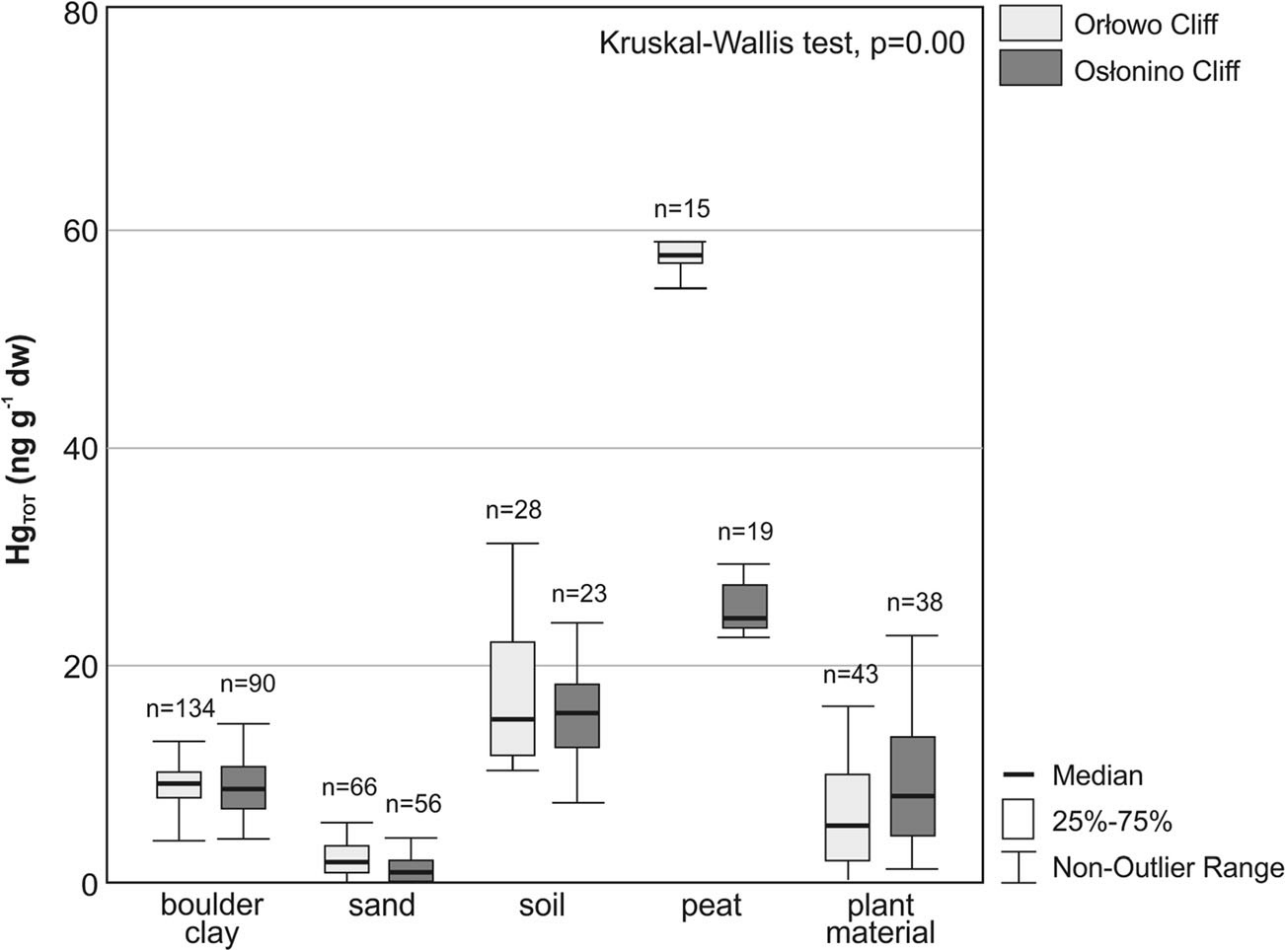
Concentration range of Hg_TOT_ (without outliers and extremes) in different types of colluvial material of Orłowo and Osłonino cliffs in 2011–2014

The Hg_TOT_ concentrations found in particular types of the study material were significantly different (Kruskal–Wallis test, *p* = 0.00) (Fig. 2). The main factor determining the level of Hg_TOT_ in the clastic material crumbling off the cliff was the type of deposit—a statistically relevant, strong positive correlation was observed between the concentration of this metal in a sample and the content of fine fraction <0.063 mm (R Spearman = 0.76) and the loss of ignition percentage in the deposits (R Spearman = 0.82). The highest Hg_TOT_ concentrations were found in peaty sediments with the obtained values ranging from 22.4 to 67.9 ng g^−1^ dw (*n* = 34, mean 40.0 ng g^−1^ dw, median 28.9 ng g^−1^ dw), which was associated with the high LOI values (over 30 %). Hg_TOT_ concentrations in the dominated type of deposits along the Polish cliff coast, the boulder clay, ranged from 4.1 to 18.2 ng g^−1^ dw (*n* = 304, mean 8.8 ng g^−1^ dw, median 8.9 ng g^−1^ dw). The lowest Hg_TOT_ concentrations occurred in sands, where they ranged between 0.1 and 5.8 ng g^−1^ dw (*n* = 145, mean 1.9 ng g^−1^ dw, median 1.5 ng g^−1^ dw), which was related mainly to the low content of fine sediment fraction (<5 %) and organic matter percentage (<3 %). Concentration of Hg_TOT_ in the plant material ranged from 0.2 to 102.6 ng g^−1^ dw (*n* = 81, mean 10.9 ng g^−1^ dw, median 7.1 ng g^−1^ dw), but only 10 % of Hg_TOT_ concentrations exceeded 50 ng g^−1^ dw (Fig. 2).

**Fig. 3:**
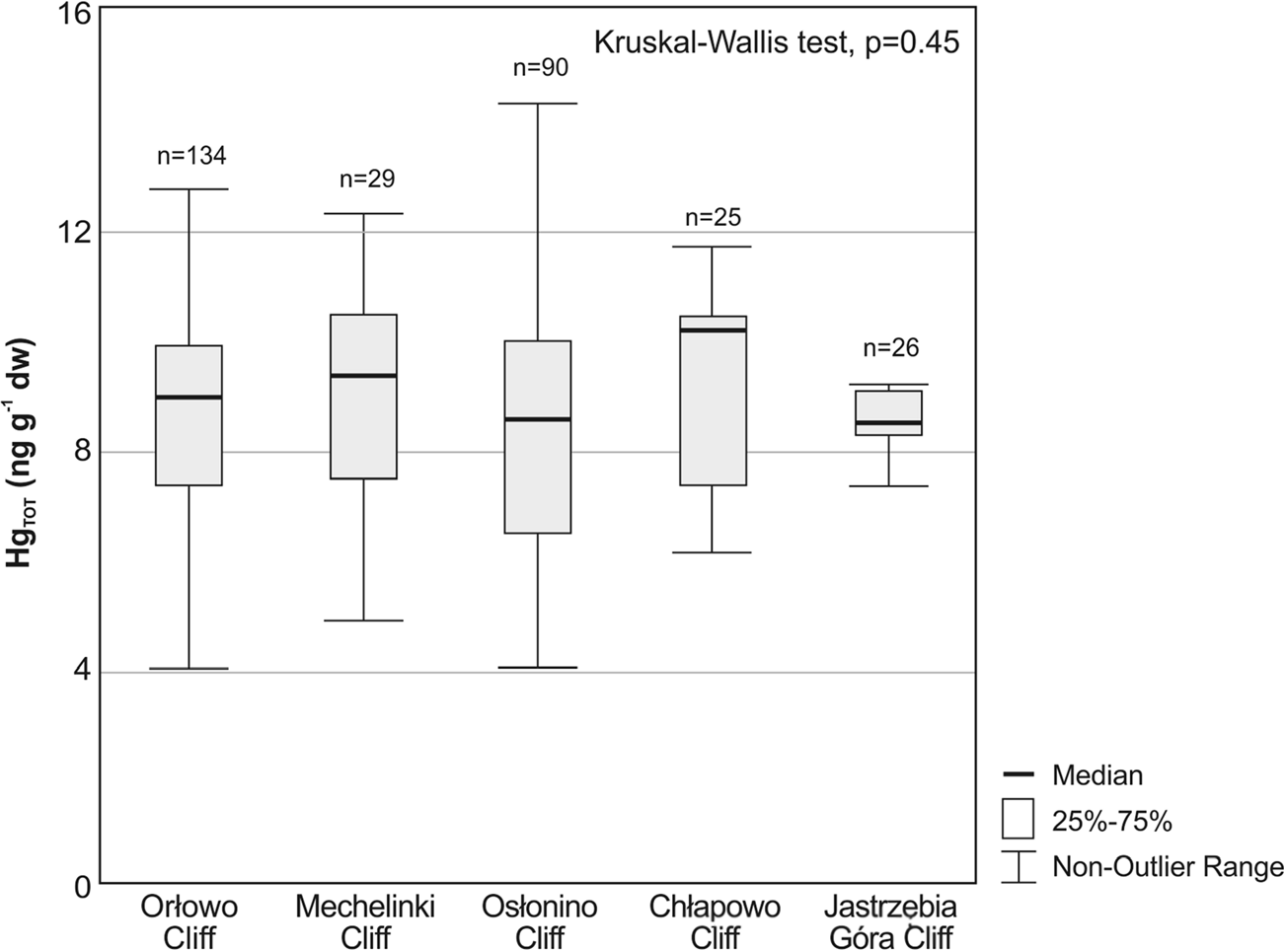
Concentration range of Hg_TOT_ (without outliers and extremes) in boulder clay from colluvial material of Orłowo, Mechelinki, Osłonino, Chłapowo and Jastrzębia Góra cliffs in 2011–2014

Hg_TOT_ concentrations in boulder clay at the five studied cliffs were within a similar range and the differences between all five cliffs were not statistically significant (Kruskal–Wallis test, *p* = 0.45). Median of Hg_TOT_ concentration varied from 8.5 ng g^−1^ dw at Jastrzębia Góra to 10.4 ng g^−1^ dw in Chłapowo (Fig. 3), while the predominant range of Hg_TOT_ level was from 8 to 11 ng g^−1^ dw, values within that range constituting 51 % of all the values obtained.

### Hg load

**Fig. 4:**
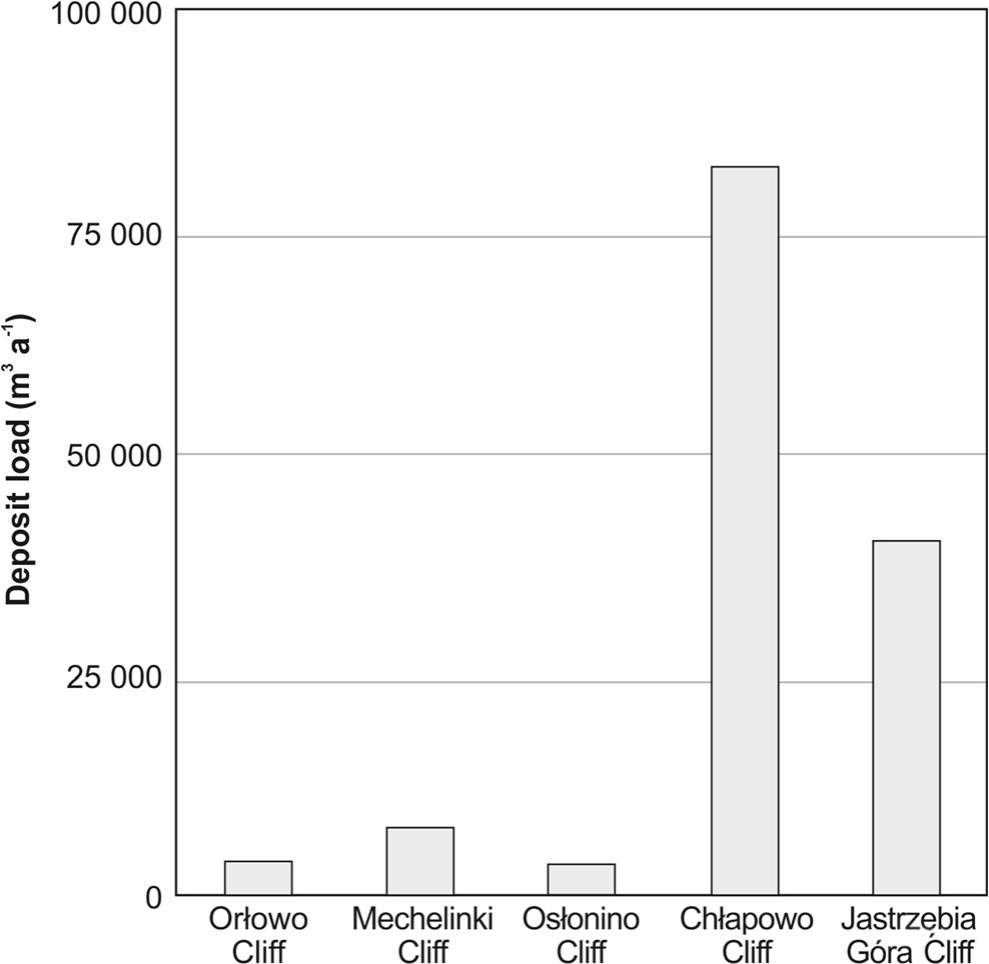
Average annual load of deposits (m^3^ a^−1^) crumbling off the Orłowo, Mechelinki, Osłonino, Chłapowo and Jastrzębia Góra cliffs in 2011–2014

The studied cliffs located at the Polish coast of the southern Baltic Sea varied in terms of height and length, but most of all in the retreat rate (Table 1). These factors determined the size of the load of deposits that was released from a given cliff through the processes of coast abrasion. According to the long-term changes (in years 1875–1979) of the analysed cliff profiles, the retreat rates varied from the 0.05 m a^−1^ at Mechelinki to the 0.61 m a^−1^ at Chłapowo (Osłonino 0.20 m a^−1^; Jastrzębia Góra 0.31 m a^−1^ Orłowo: 0.50 m a^−1^) (Zawadzka-Kahlau 1999). Results obtained from the airborne LiDAR indicate the annual loss of the deposits was the highest at cliffs located in the area of the open sea—Chłapowo (82,360 m^3^ a^−1^ = 218,254 t a^−1^) and Jastrzębia Góra (40,045 m^3^ a^−1^ = 106,119 t a^−1^). The loads of sedimentary material introduced into the marine environment due to erosion of the cliffs located at the Gulf of Gdańsk coast—Osłonino (3236 m^3^ a^−1^ = 8575 t a^−1^), Orłowo (3640 m^3^ a^−1^ = 9647 t a^−1^) and Mechelinki (7576 m^3^ a^−1^ = 20,076 t a^−1^)—were many times lower than in the open part of the coast (Fig. 4, Table 1). This is mainly related to the length the cliffs—Jastrzębia Góra (length 3.95 km) and Chłapowo (length 2.80 km)—constituting far longer section of the coast in comparison to the cliffs located in the Gulf of Gdańsk (the length of each cliff is less than 1 km). In addition, an increased loss of sediment in the area of open sea is associated with a much more dynamic environment of the coastal zone (i.e. stronger currents, waves, often occurring storms) than in the western part of the Gulf of Gdańsk (Mojski 2000).

A total volume of deposits crumbling of the analysed cliffs (summary length 8.1 km) was 136,857 m^3^ a^−1^ with a total weight of 362,672 t. In the case of the cliffs located on the coast of the open Baltic (total length 6.8 km), those at Chłapowo and Jastrzębia Góra, the abrasion processes were jointly responsible for the release of 122,403 m^3^ a^−1^ of sedimentary material, weighing 324,373 t (Table 1, Fig. 4). The contribution of the Chłapowo cliff to this load was 67 %, while Jastrzębia Góra contributed 33 %. At the cliffs located in the coast of the Gulf of Gdańsk (total length 1.3 km), those at Orłowo, Mechelinki and Osłonino, almost 14,500 m^3^ a^−1^ of total sedimentary material with a mass of over 38,000 t was introduced into the basin over the course of 1 year as a result of coastal abrasion (Table 1, Fig. 4). Over half (53 %) of this mass was constituted by sediment released as a result of cliff abrasion in Mechelinki, while the amounts of deposits from the Osłonino and Orłowo cliffs account for the remaining 22 and 25 %, respectively.

Due to the fact that the concentration of Hg_TOT_ in the boulder clay, the predominant structural component of the cliffs (Subotowicz 1980), from the five analysed regions of the Polish shore, did not significantly differ (Fig. 3) for calculating the Hg load, the mean value of the Hg_TOT_ was used. Taking into account the Hg_TOT_ concentration in the deposits (8.8 ng g^−1^ dw) and the total mass of the released sediment (362,672 t), the calculated Hg load introduced to the marine environment within 1 year amounted to 3.2 kg (Table 1) (example of Hg load calculation was included in the Online Resource 2). The combined length of these cliffs is 8.1 km; therefore, it can be estimated that the 1-km long section of the cliff is responsible for introduction to the sea of the Hg load reaching 0.4 kg.

**Fig. 5:**
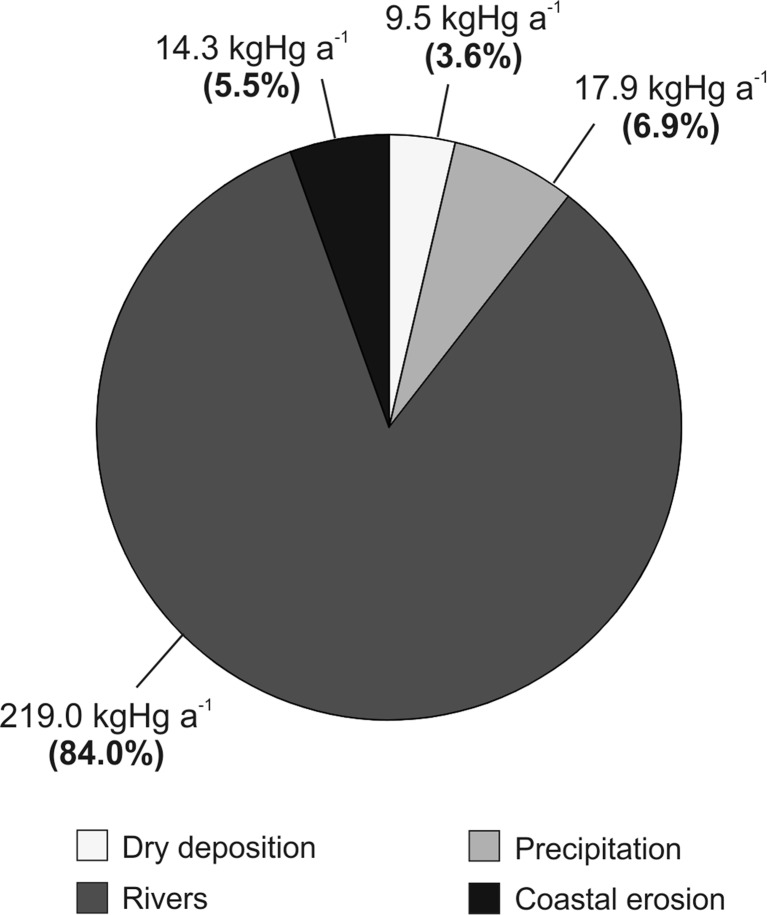
Impact of coastal erosion on the Hg load into the Gulf of Gdańsk (southern Baltic) compared to riverine input and atmospheric deposition in 2011–2014 (dry deposition data based on Saniewska 2013)

Based on the information that the erosion section of the shore of the Gulf of Gdańsk is 36 km long, the annual Hg load introduced to the basin through the abrasion of the gulf’s coast can be estimated at 14.3 kg. Taking into account the monthly measurements of Hg_TOT_ concentrations conducted in rivers and the flow of each river (Vistula: mean concentration 7.4 ng Hg L^−1^, mean flow 774 m^3^ s^−1^; Reda: mean concentration 6.4 ngHg L^−1^, mean flow 7.4 m^3^ s^−1^; Gizdepka and Kacza: mean concentration 6.0 ng Hg L^−1^, mean flow 0.2 m^3^ s^−1^), the inflow of Hg via rivers into the Gulf of Gdańsk was estimated for the year 2012: via the Vistula 218 kg a^−1^, the Reda 1.0 kg a^−1^, while the smaller rivers introduced about 39 g Hg a^−1^ each. The combined Hg inflow in 2012 was 219 kg, which means that the inflow of the Hg through coastal abrasion (14.3 kg) equalled 6.5 % of the river inflow for that year. The amount of Hg that was introduced into the Gulf of Gdańsk with precipitation in 2012 was 17.9 kg (mean Hg_TOT_ concentration in rains 4.8 ng L^−1^, total precipitation 599 mm). Here, coastal abrasion load represents 80 % of the total Hg inflow with rains for the whole of 2012. In the case of the dry deposition, the Hg load in the Gulf of Gdańsk region has been estimated at 9.5 kg a^−1^ (Saniewska 2013). This value is 50 % lower compared with the Hg load introduced to the gulf due to erosion of the coast calculated by the authors. These calculations allowed to state that coastal erosion is responsible for over 5 % of the Hg load in the Gulf of Gdańsk and it is the third most important source of Hg to the gulf, after rivers and rainfall (Fig. 5).

According to published data, about 147 km of the Polish coastline is undergoing intensive erosion (Uścinowicz et al. 2004). Taking into account the mean Hg_TOT_ concentration assayed in boulder clay (8.8 ng g^−1^ dw), the yearly Hg load that can be introduced to the southern Baltic as a result of cliff abrasion along the Polish coastline is 58.3 kg in total. This value equals 27 % of the load introduced to the Baltic with the Vistula in 2012, the second largest river flowing out into the Baltic Sea (after the Neva River) (HELCOM 2004). For the whole of the Baltic, due to the existence of soft-rock cliffs (which are prone to abrasion) in many areas such as in Germany, Lithuania, Latvia or Estonia, this value can be many times higher (Soomere et al. 2011).

The calculation of the Hg load took into account only mercury concentration in boulder clay, it being the main structural material of the cliffs in the area of the southern Baltic. Based on the results of the study on the ALS method sensitivity obtained by Earlie et al. 2013 at the cliff coast of United Kingdom in 2007–2011, the average errors from the LiDAR technology accounted for 20 %, while the smaller were the changes, the larger was the error. The error, in the sections of the coast where the abrasion rate was most similar (ca. 0.5 m a^−1^) to the Polish coast, was relatively low and ranged from 3 to 10 % (Earlie at al. 2013). The calculated Hg load did not include the Hg-rich peats and soils or the plant material (Fig. 2), owing to the fact that their mass was impossible to assess. This means that these data may be significantly underestimated, and coastal erosion may constitute an even greater share in Hg load to the marine environment.

What is more, the consequences of extreme natural phenomena, being a considerable factor responsible for the crumbling of sizeable masses of sediment in the region of cliff coastline. According to forecasts, higher frequency and intensity of extreme phenomena may increase the rate of coast erosion by 80 % on average and will affect more than 75 % of the total length of the Polish coastline. In addition to this, western circulation, typical for this part of the southern Baltic, will increase the off-coast transport of sediments, making the sea coast less resilient and accelerating its erosion (HELCOM 2013). Such changes have been observed, for example by Dubrawski and Zawadzka-Kahlau (2006) at the end of 2001 to the beginning of 2002. As shown by studies into the change processes of the shape of the coastline in the last few years, when the influence of storms is particularly strong, the speed of abrasion can be several times higher than long-term means. This is exemplified by the Jastrzębia Góra cliff section, where the coastline receded by more than 19 m in the years 2007–2011 (abrasion rate 2.7 m a^−1^—over twice as high as the annual mean) (Kamiński 2012).

Unlike the load of pollutants introduced via rivers, which is distributed throughout the year, the Hg load from crumbling land is introduced by impulses, within a relatively short time. In the immediate aftermath of a storm, this resulted in an increase in Hg_TOT_ concentration which was as much as ten times higher than that of the preceding month (up to the value of 33 ng L^−1^, annual mean for 2012 amounted to 3.7 ng L^−1^) in the seawater close to the Orłowo cliff. In the Osłonino cliff area, where water exchange is very limited (Saniewska et al. 2010), the concentration of Hg_TOT_ immediately after a storm reached as high as 62 ng L^−1^. This value was more than 30 times higher than the concentration from 3 weeks earlier (annual mean for 2012 was 3.8 ng L^−1^). This is of particular importance to marine organisms living in this part of the coastal zone, as well as for people who catch fish and seafood in the areas where coastal abrasion takes place. Studies carried out in 2012 in the area of the Orłowo cliff, where, for safety reasons, a post-war bunker was knocked off the top of the cliff, show a significant change in the cliff profile and a considerable loss of sediments (Janowski et al. 2014). Analyses of Hg concentrations in the coastal zone at the base of the cliff before and 7 days after the removal of the bunker showed a 2.5-fold increase in the concentration of Hg in suspended matter and a 10-fold increase of Hg concentration in phytoplankton (Bełdowska 2015). This confirms the potential influence of coastal erosion on the inflow of toxic mercury to the marine trophic chain. Moreover, the preliminary results of parallel research conducted by the authors in the coastal zone of the Gulf of Gdańsk indicate that fine-grained sedimentary material derived from coastal erosion can be a significant source of mercury in phytobenthos as well as zoobenthos feed on suspended matter (Jędruch et al. 2013; Bełdowska et al. 2014a).

## Summary

Nowadays, thanks to our wider knowledge of Hg toxicity, its input into the marine environment has been limited. However, human activity is not its only means of introduction into the Baltic Sea. The extreme natural phenomena such as floods contribute to the introduction of significant amounts of material from land to the seas (Saniewska et al. 2014b). Another process that affects the entrance of Hg to the marine environment is coastal erosion. Coastal zone abrasion in the Gulf of Gdańsk raises the estimated Hg inflow over 5 % and presents third in importance source of Hg, after rivers and precipitation. In comparison to the main source of Hg which is rivers, the contribution of coastal erosion appears to be inconsiderable. What should however be noted is that 99.5 % of riverine input of Hg to the Gulf of Gdańsk is introduced by the Vistula—the second largest river in the Baltic Sea drainage basin. In comparison to the small, local rivers (Reda, Kacza and Gizdepka), the importance of coastal erosion, in turn, is more meaningful. Taking into account the total annual load of these rivers (1.04 kg), the coastal erosion introduces into the marine environment over 13 times more Hg. It means that the process of abrasion may be particularly significant in areas away from the mouths of large rivers or in bays without any major river outlets, being an important Hg source in the coastal zone. This may be even more relevant when, at the same time, the agricultural fields and forest soils are eroded.

The results presented in this study also indicate that coastal erosion should be included in the Hg cycle in the environment and taken into account by institutions and researchers calculating the Hg loads and budget, especially in the marine coastal zone. So far, this source was not taken into account either in local (i.e. Wrembel 1993; Saniewska 2013) or wider scale (i.e. Matschullat 1997; Szefer 2002; HELCOM 2010), which means that the presented loads had been underestimated. Coastal erosion may be important source of Hg especially in the area of occurrence of easily erodible soft-rock cliffs, where a large Hg load of mercury could enter the coastal zone within a short time. Hg getting into the marine environment during a single event of crumbling cliff could endanger organisms living there and be more harmful than a constant flow of metal via rivers during the year. Since the concentration of mercury in the cliff deposits entering the coastal zone is similar to the level of natural background, due to their large mass, this problem is significant.

## Electronic supplementary material

Below is the link to the electronic supplementary material.

Online Resource 1 (PDF 455 kb)

Online Resource 2 (PDF 333 kb)
