# Supplementary material for: Coastal erosion as a source of mercury into the marine environment along the Polish Baltic shore
Source: Environ Sci Pollut Res Int. 2016 May 10;23:16372–82. doi: 10.1007/s11356-016-6753-7 (PMC4975767; doi:10.1007/s11356-016-6753-7)

Online Resource 1

Supplementary material to the article published in Environmental Science and Pollution Research

Coastal erosion as a source of mercury into the marine environment along the Polish Baltic shore

by M. Bełdowska, A. Jędruch (✉), L. Łęczyński, D. Saniewska and U. Kwasigroch
University of Gdansk, Institute of Oceanography, Pilsudskiego 46, 81-378 Gdynia, Poland
✉ Corresponding author e-mail: agnieszka.jedruch@ug.edu.pl

Map of the cliff area showing the investigated sector (Polish coastline section: 81.30 km – 81.95 km) based on the Airborne Laser Scanning (ALS) results (Orłowo Cliff example):

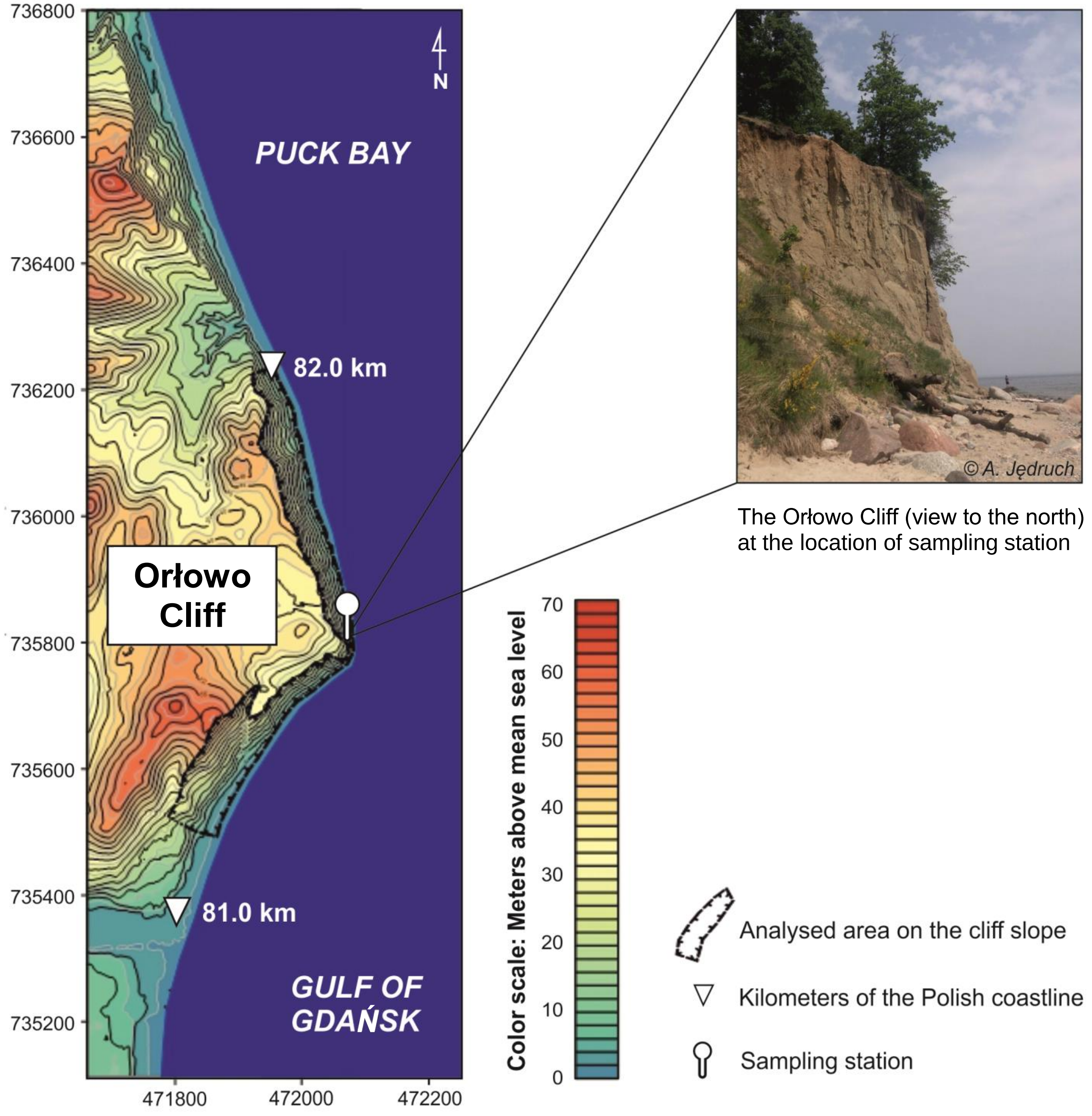

Supplement: Supplementary file 1 — (PDF 455 kb) [file 11356_2016_6753_MOESM1_ESM.pdf]
