# Supplementary material for: Coastal erosion as a source of mercury into the marine environment along the Polish Baltic shore
Source: Environ Sci Pollut Res Int. 2016 May 10;23:16372–82. doi: 10.1007/s11356-016-6753-7 (PMC4975767; doi:10.1007/s11356-016-6753-7)
Supplement: Supplementary file 2 — (PDF 333 kb) [file 11356_2016_6753_MOESM2_ESM.pdf]

Online Resource 2

Supplementary material to the article published in Environmental Science and Pollution Research
Coastal erosion as a source of mercury into the marine environment along the Polish Baltic shore
by M. Bełdowska, A. Jędruch (✉), L. Łęczyński, D. Saniewska and U. Kwasigroch
University of Gdansk, Institute of Oceanography, Pilsudskiego 46, 81-378 Gdynia, Poland
✉ Corresponding author e-mail: agnieszka.jedruch@ug.edu.pl

Temporal changes in the active layer of deposits occurring on the surface of the cliff slope based on the Airborne Laser Scanning (ALS), LiDAR results (Orłowo Cliff example):

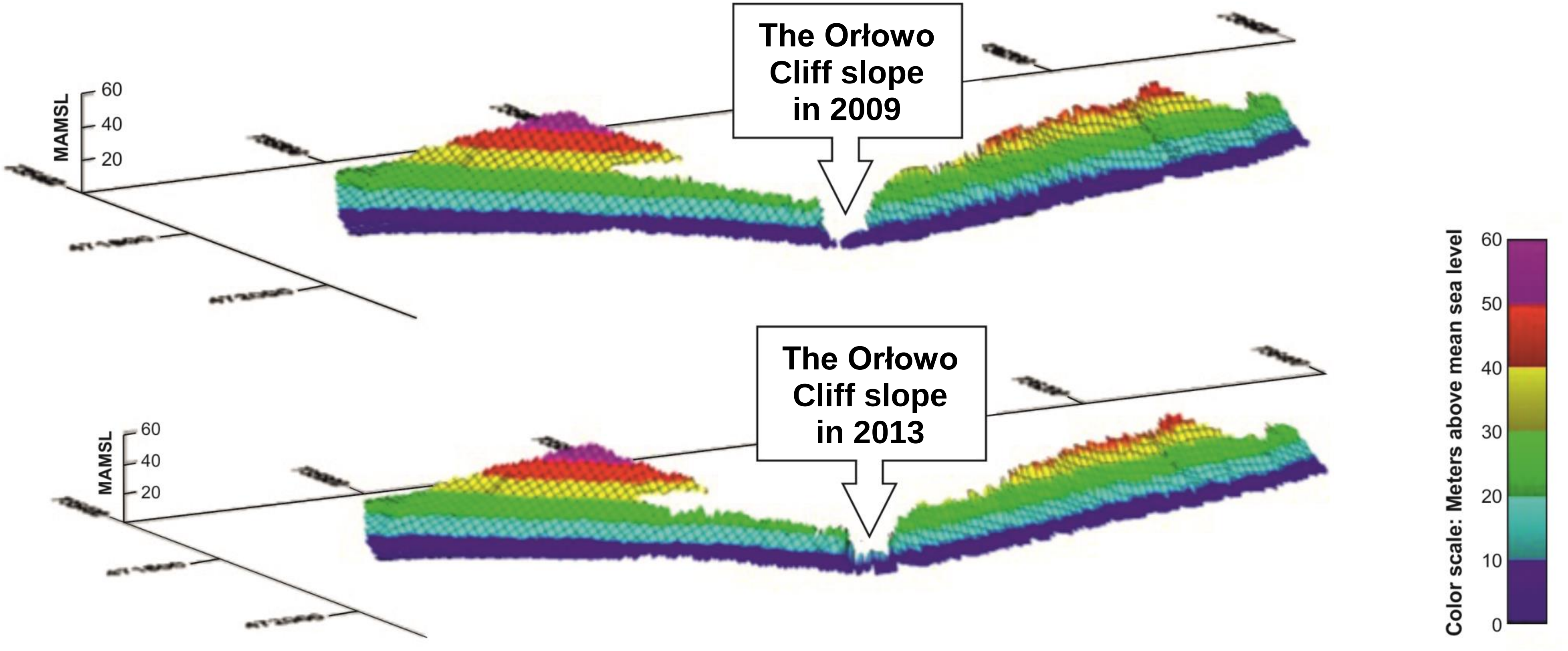

Determination of the Hg load introduced into the marine environment due to coastal erosion (Orłowo Cliff example):

The volume difference between Orłowo Cliff slopes in years 2009 and 2013: **14 561 m^3**
The annual average volume of sedimentary material loss: **3 640 m^3 a^{-1}**
The annual average mass of sedimentary material loss: **9 647 t a^{-1}** (the sediment density used to the calculation: **2.65 g cm^{-3}**)
The annual Hg load**: 0.08 kg a^{-1}** (the Hg level in the deposits used to the calculation: 8.8 ng g^{-1} dw)
